# Validated and enriched Android application dataset: integration of VirusTotal and Quark-Engine intelligence

**DOI:** 10.1016/j.dib.2026.113152

**Published:** 2026-08-11

**Authors:** Fadi Mohsen, Yulin Zhang, Ali Murat Gültekin, Halil Bisgin, Koerner Buchta

**Affiliations:** a University of Groningen, Groningen, the Netherlands; b Duke University, Durham, NC, USA; c Sabanci University, Istanbul, Turkey; d University of Michigan, Ann Arbor, MI, USA

**Keywords:** Threat intelligence, APK validation, Malware intelligence, Data curation, Reproducible benchmarking, Android application security

## Abstract

This data article describes a large-scale Android application dataset built by enriching a previously released Google Play removal corpus with external malware intelligence from VirusTotal Premium v3 Enterprise and Quark-Engine 25.3.1. The source corpus contains 870,514 applications with 48 metadata features. Of these, 803,320 applications (92.3%) were matched to VirusTotal reports, 772,706 (88.7%) were successfully parsed from Quark-Engine scans, and 772,506 (88.7%) had paired analyses from both sources (with an additional 27 applications having VirusTotal data only, included in the summary dataset). The released paired dataset combines the original metadata with ten selected derived features—five from VirusTotal and four from Quark-Engine—yielding 58 analytical features per application, or 59 columns when the MD5 linkage key is included. The release contains processed extensive tables, a compact summary dataset, and five consensus-based validated subsets whose sizes range from 38,302 to 80,368 applications depending on the VirusTotal threshold.

Specifications Table

**Table:** 

Subject	Computer Sciences
Specific subject area	Android benign and malicious datasets, threat-intelligence enrichment, and dataset validation for reproducible benchmarking
Type of data	CSV files
Data collection	VirusTotal Premium v3 Enterprise reports were retrieved by MD5 hash via the v3 API from March to May 2025. Quark-Engine 25.3.1 was executed locally on APK files using a two-stage timeout policy: 2 min initially, then 10 min for previously skipped APKs. Parsed JSON outputs were normalized (numeric missing = −1, categorical missing = None) and merged by MD5 hash. The source corpus is the Google Play removal dataset published by Mohsen et al. (2022).
Data source location	Institution: University of Groningen; City: Groningen; Country: The Netherlands. Primary source data: Google Play application metadata and APK-derived manifest features released by Mohsen et al. [1,2].
Data accessibility	Repository name: DataverseNL Data identification number: DOI: 10.34894/XV84Z8 Direct URL to data: https://doi.org/10.34894/XV84Z8 Instructions for accessing these data: No registration or access request required. All files are publicly available under CC BY 4.0 license. Processing scripts are publicly available at: https://github.com/BisginLab/DataInBrief-VT-QE
Related research article	None at the time of submission. If the companion research article is accepted before proof stage, this row will be updated with its final citation in the journal's required format.

## 1 Value of the Data

• Why these data are valuable: Unlike single-source Android malware datasets (e.g., DREBIN, AndroZoo), this corpus integrates three independent labels per application: original Google Play removal status, VirusTotal multi-engine consensus, and Quark-Engine static analysis. This three-source design enables researchers to study label disagreement, quantify label noise, and build more robust ground-truth benchmarks. The transparent reporting of coverage gaps (11.3% unrecovered), missingness rates, timeout behavior (42.7% larger files timed out), and parsing failures (27 apps skipped) allows users to assess fitness for their specific use case.
• How these data can be reused for benchmarking: Five pre-validated benchmark subsets are provided at VirusTotal thresholds k = 1 through k = 5. Each subset requires consensus among all three label sources, giving users a principled way to trade dataset size (38,302 to 80,368 samples) against label strictness. Researchers can select the threshold that matches their experimental design without re-implementing validation logic. The subsets are ready for immediate use in malware detection evaluation, classifier comparison, and label-noise sensitivity analysis.
• How the data can be reused for feature engineering: The extended paired dataset combines the original 48 metadata features with nine derived security features (five from VirusTotal, four from Quark-Engine), yielding 59 analytical columns per application linked by MD5 hash. Researchers can extract subsets for feature selection studies, correlation analysis, or input to machine learning pipelines without running months of API queries or local scans. All derived features have documented definitions and completeness rates (e.g., 95.372% of paired apps contain all five VirusTotal features).
• Why transparency supports reproducible research: The release documents collection coverage at each pipeline stage (VirusTotal: 92.3%, Quark-Engine: 88.8%, paired: 88.7%), missing field distributions (Fig. 4), and cross-source agreement patterns (Fig. 6). This transparency allows other researchers to reproduce the enrichment pipeline, compare against future collections, or meta-analyze tool behavior over time. Unlike proprietary or access-controlled datasets, this corpus is publicly available under CC BY 4.0 license with persistent DataverseNL DOI.
• How these data support label-noise research: Android malware research has long struggled with label quality, as VirusTotal verdicts drift over time and single-threshold rules produce inconsistent ground truth. By providing consensus-based labels and documenting disagreement between sources (e.g., 86.7% of Quark Moderate Risk apps have zero VirusTotal detections), this dataset enables systematic study of label noise sources, threshold sensitivity, and ensemble labeling strategies. Researchers can use the provided subsets to benchmark noise-mitigation algorithms.
• Concrete usage example: A researcher seeking a balanced binary classification benchmark would select the k = 5 validated subset (19,275 benign; 19,027 malicious), while a researcher prioritizing dataset size over label strictness would select the k = 1 subset (80,368 samples). This flexibility allows users to match the dataset to their specific experimental design without re-implementing validation logic.
• Class imbalance note: Users should note that the validated subsets exhibit class imbalance (e.g., 18,275 benign vs. 62,093 malicious at k = 1). See Limitations for mitigation recommendations, including stratified sampling and cost-sensitive learning.

## 2 Background

This dataset extends the previously published Google Play removal corpus introduced by Mohsen et al. [1,2]. Large Android app repositories such as AndroZoo [3] have made large-scale benchmarking feasible, but prior work has shown that labels inferred from multi-scanner services can drift over time [4] and can be sensitive to threshold-based rules [5,6]. More recent work has therefore emphasized label-noise identification and mitigation in Android malware research [7,8]. In parallel, rule-based analysis frameworks such as Quark-Engine provide interpretable code-level signals that complement reputation-oriented threat intelligence [9]. Within that context, the present data article documents how VirusTotal and Quark-Engine outputs were collected, normalized, linked to the source corpus, and converted into reusable tables and validated subsets. The emphasis here is on the released data products, schema, provenance, coverage, and validation rules rather than on classifier performance claims.

## 3 Data Description

The public release contains five linked dataset components:

1. VirusTotal extensive dataset parsed from raw VirusTotal JSON responses.
2. Quark-Engine extensive dataset parsed from raw Quark-Engine JSON responses.
3. Summary dataset containing MD5 and the nine selected derived features.
4. Extended paired dataset combining the original metadata with the nine selected VirusTotal and Quark-Engine features.
5. Validated subsets generated for VirusTotal thresholds (k=1,…,5).

All components are linked by MD5 hash.

### 3.1 Dimensions of the released datasets

Table 1 reports the dimensions of the main released tables. For the validated subsets, the exact dimensions are reported separately in Table 3 because they vary with the chosen VirusTotal threshold Table 4.

**Table 1:** Dimensions and purpose of the released dataset components.

Dataset version	Rows × columns	MD5 key	Purpose
Original Google Play metadata corpus	870,514 × 48	Associated source key	Baseline metadata corpus from the original release [2]
VirusTotal extensive table	803,320 × 55	Included	Parsed threat-intelligence attributes from VirusTotal JSON responses
Quark-Engine extensive table	772,706 × 12	Included	Parsed risk and per-rule evidence fields from Quark-Engine JSON responses
Summary dataset	772,533 × 11	Included	Compact MD5 + 9 feature table for rapid analysis†
Extended paired dataset	772,506 × 59	Included	Original metadata + 9 derived security features†^,^‡
Validated subsets (k=1,…,5)	See Table 4	Included	Consensus-clean benchmark datasets

† The aggregated_risk_score feature, included in the summary and extended paired datasets, is constant (211) across all samples and provides no discriminative power. It is retained in the dataset only to avoid creating a new version. Users should omit it from downstream modeling.

‡ The Summary dataset (772,533 rows) includes 27 applications with complete VirusTotal data but null Quark-Engine values. These 27 applications are excluded from the Extended paired dataset (772,506 rows) and the validated subsets.

### 3.2 Selected derived features

VirusTotal features

1. malicious_count — number of engines labeling the APK as malicious.
2. undetected_count — number of engines labeling the APK as undetected.
3. certificate_life_days — validity period of the signing certificate in days.
4. file_duration_days — time span between first and last submission in VirusTotal.
5. times_submitted — number of times the APK was submitted to VirusTotal.

Quark-Engine features

1. threat_level — Quark-Engine risk level (low, moderate, high).
2. weighted_conf_sum — confidence-weighted sum of crime weights.
3. permission_n — number of permission-related crimes.
4. avg_weight — average severity weight across detected crimes.

### 3.3 Coverage and descriptive statistics

**Fig. 1:**
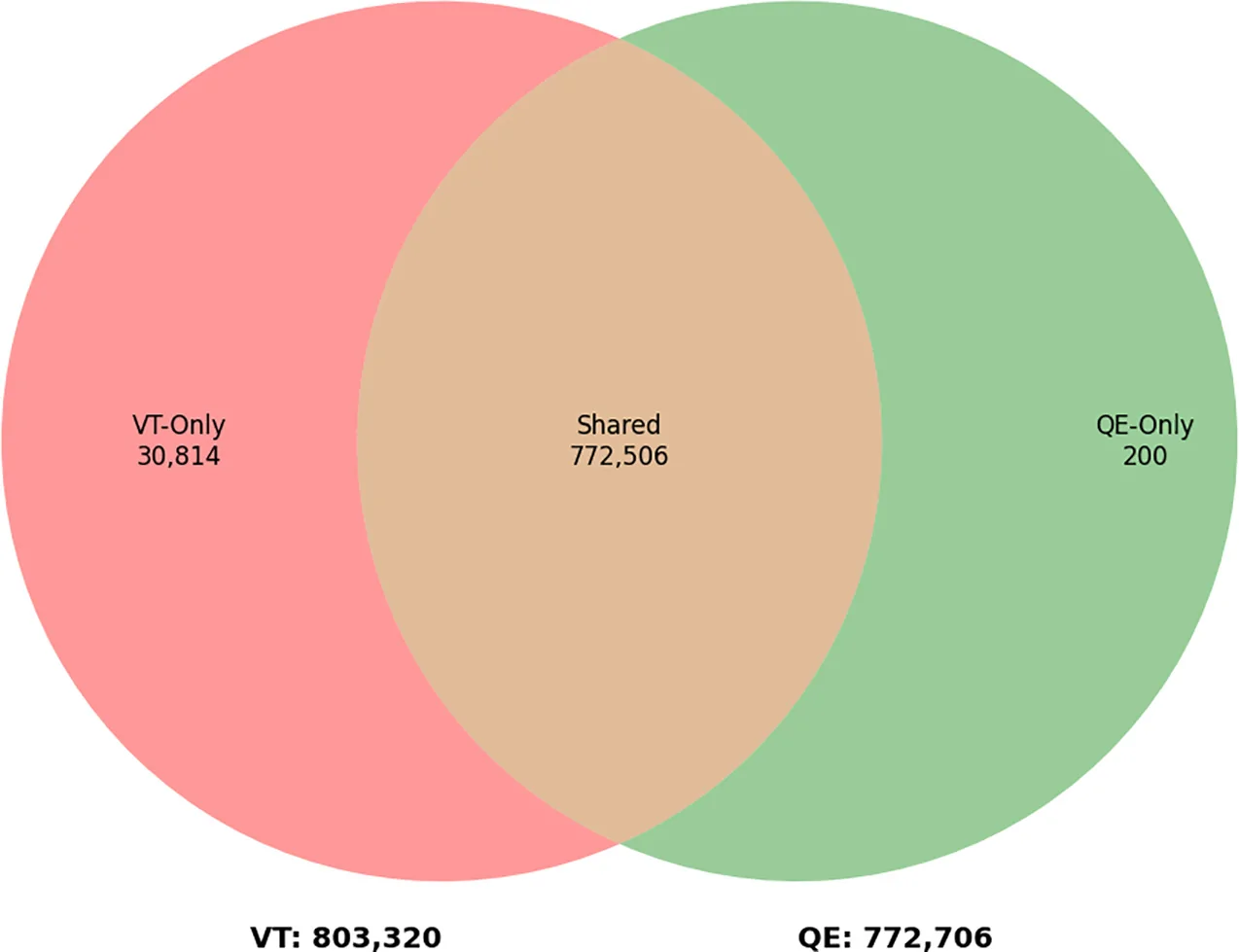
Coverage overlap of unique MD5 hashes in the parsed VirusTotal and Quark-Engine outputs. VirusTotal contributes 803,320 unique MD5s, Quark-Engine contributes 772,706, the shared intersection is 772,506, VirusTotal-only coverage is 30,814 hashes, and Quark-Engine-only coverage is 200 hashes.

Table 2 summarizes the main collection stages. Fig. 1 shows the overlap of unique MD5 hashes in the parsed VirusTotal and Quark-Engine outputs. Because that figure is based on unique parsed MD5s, its totals differ slightly from the raw report counts in Table 2.

**Table 2:** Coverage of the enrichment pipeline.

Pipeline stage	Records	Coverage of original	Notes
Original dataset	870,514	100.0%	48 metadata features from the original study [1]
VirusTotal reports	803,320	92.3%	Retrieved through VirusTotal Premium v3 Enterprise MD5 lookups [10]
Quark-Engine reports	772,706	88.7%	Local scans produced by Quark-Engine 25.3.1 [9]
Summary dataset	772,533	88.7%	Includes 27 apps with VT data but null QE values
Paired VT+QE intersection	772,506	88.7%	Final paired set used for summary and extended datasets
Unavailable after pairing	98,008	11.3%	Missing reports, unavailable files, timeouts, or unprocessed APKs

Data count clarification. Throughout this paper, several related but distinct counts appear. For clarity:

○ 870,514: Original corpus after removing one duplicate entry during normalization.
○ 803,320: VirusTotal reports successfully retrieved.
○ 772,732: Raw Quark-Engine reports produced.
○ 772,706: Valid Quark-Engine reports after removing 26 JSON parsing failures (0.0034%).
○ 772,506: MD5 intersection of VirusTotal and valid Quark-Engine reports (the extended paired dataset).
○ 772,533: Summary dataset rows, which include 27 applications with VirusTotal data but null Quark-Engine values.

All numbers reported in tables and figures are consistent with these definitions. The validated subsets (Table 3) are derived from the extended paired dataset (772,506 applications).

**Table 3:** Class composition and dimensions of the validated subsets for different VirusTotal thresholds.

k	Benign	Malicious	Total rows	Rows × columns	Retained from 473,668 valid apps
1	18,275	62,093	80,368	80,368×59	17.0%
2	19,174	34,169	53,343	53,343×59	11.3%
3	19,225	26,628	45,853	45,853×59	9.7%
4	19,246	22,193	41,439	41,439×59	8.7%
5	19,275	19,027	38,302	38,302×59	8.1%

**Table 4:** Descriptive statistics for the nine derived security features based on the summary dataset (n = 772,533 ‡ applications).

Feature	Mean	Median	Std Dev	Min	Max
malicious_count	0.52	0	2.30	0	47
undetected_count	59.45	60	4.56	10	73
certificate_life_days (days)	41,748.82	10,000	107,415.19	−242*	2917,192
file_duration_days (days)	1359.81	1172	1030.34	0	5048
times_submitted	5.54	2	228.98	1	77,343
weighted_conf_sum	36.13	36.01	16.28	2.54	118
permission_n	4.04	4	1.31	0	8
avg_weight	0.23	0.24	0.08	0.06	0.61

⁎ Negative values indicate certificate validity period start date after end date, likely due to malformed certificates or parsing errors. These 242 samples were excluded from analyses requiring certificate age.

‡ The summary dataset includes 27 applications (0.0035%) that have complete VirusTotal data but null Quark-Engine values. These applications are excluded from the extended paired dataset (n = 772,506) and the validated subsets.

**Fig. 2:**
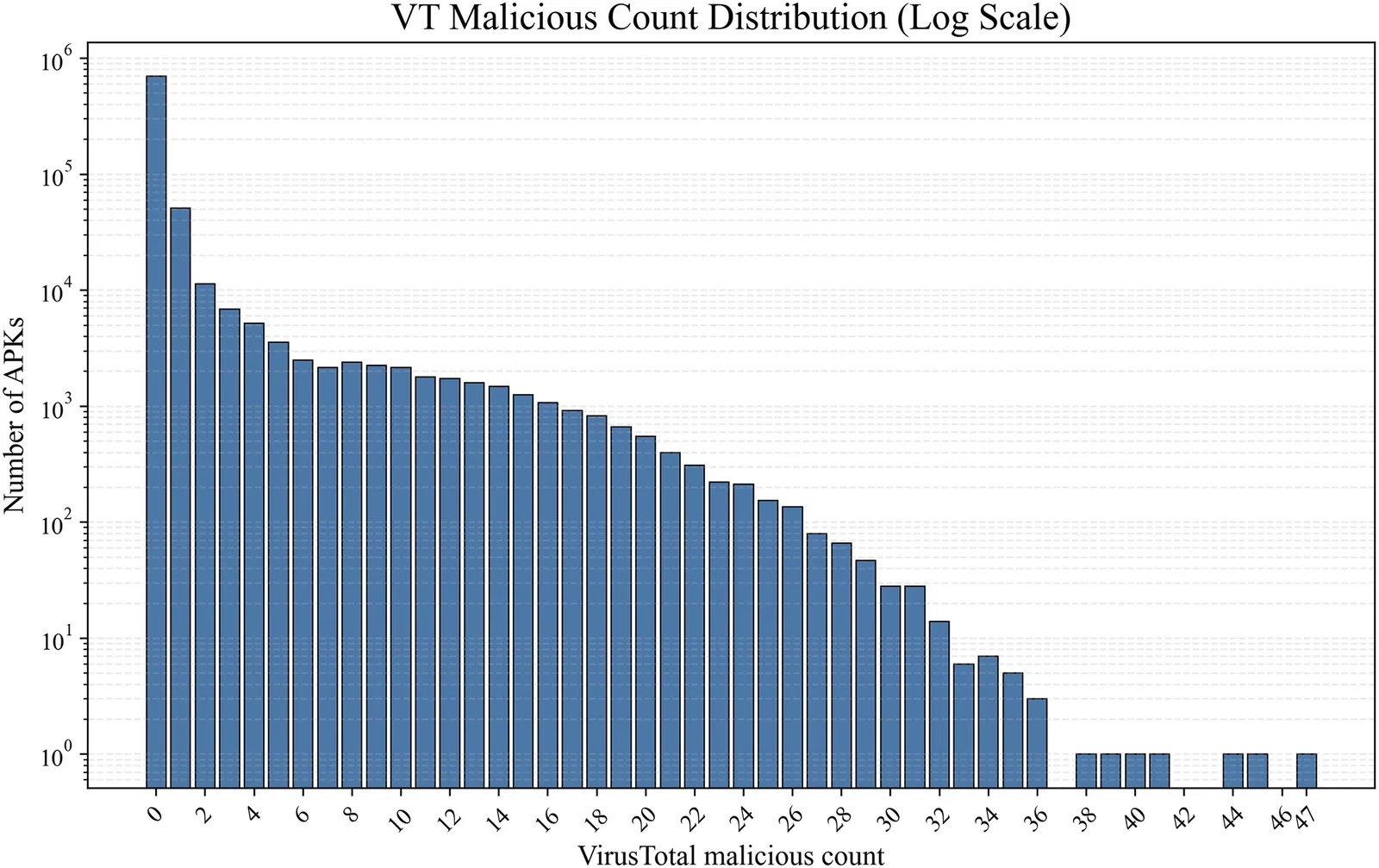
Distribution of VirusTotal malicious-engine counts on a log scale. The distribution is strongly right-skewed: 699,849 of 803,320 APKs (87.1%) have zero malicious detections, followed by 51,313 with one detection and 11,416 with two detections, with a long tail extending to 47.

Fig. 2 shows the distribution of last_analysis_stats.malicious in the VirusTotal extensive dataset. Most APKs have zero malicious detections, while a much smaller fraction accumulates agreement from multiple antivirus engines.

**Fig. 3:**
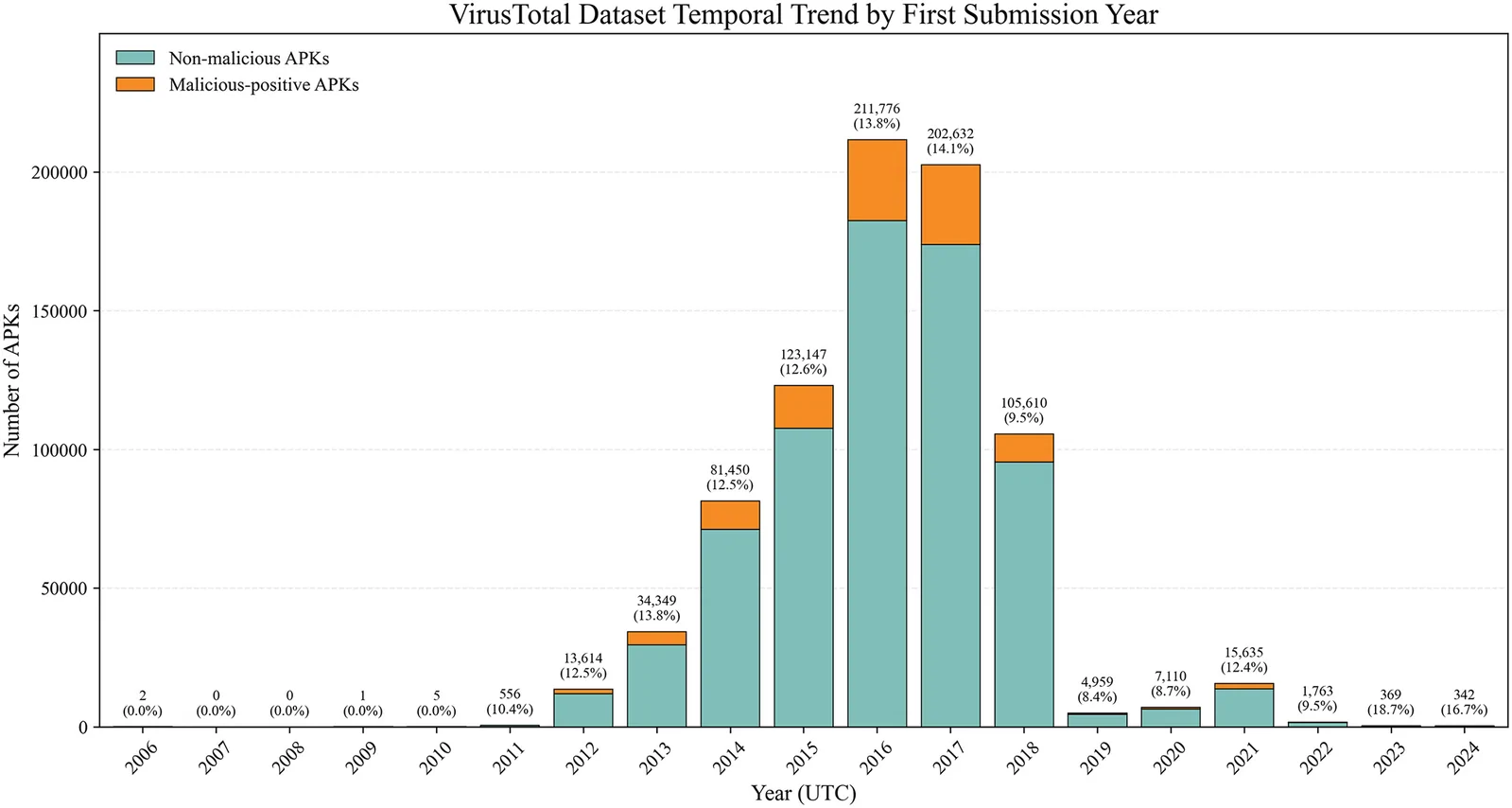
Temporal trend of the VirusTotal dataset by first submission year. The volume peaks in 2016 (211,776 APKs) and 2017 (202,632 APKs). In the main years, the malicious-positive proportion remains fairly stable, for example 12.6% in 2015, 13.8% in 2016, 14.1% in 2017, and 9.5% in 2018.

Fig. 3 summarizes yearly submissions by VirusTotal first-submission date. Peak years are 2016 and 2017, and the malicious-positive share stays relatively stable in the main years.

**Fig. 4:**
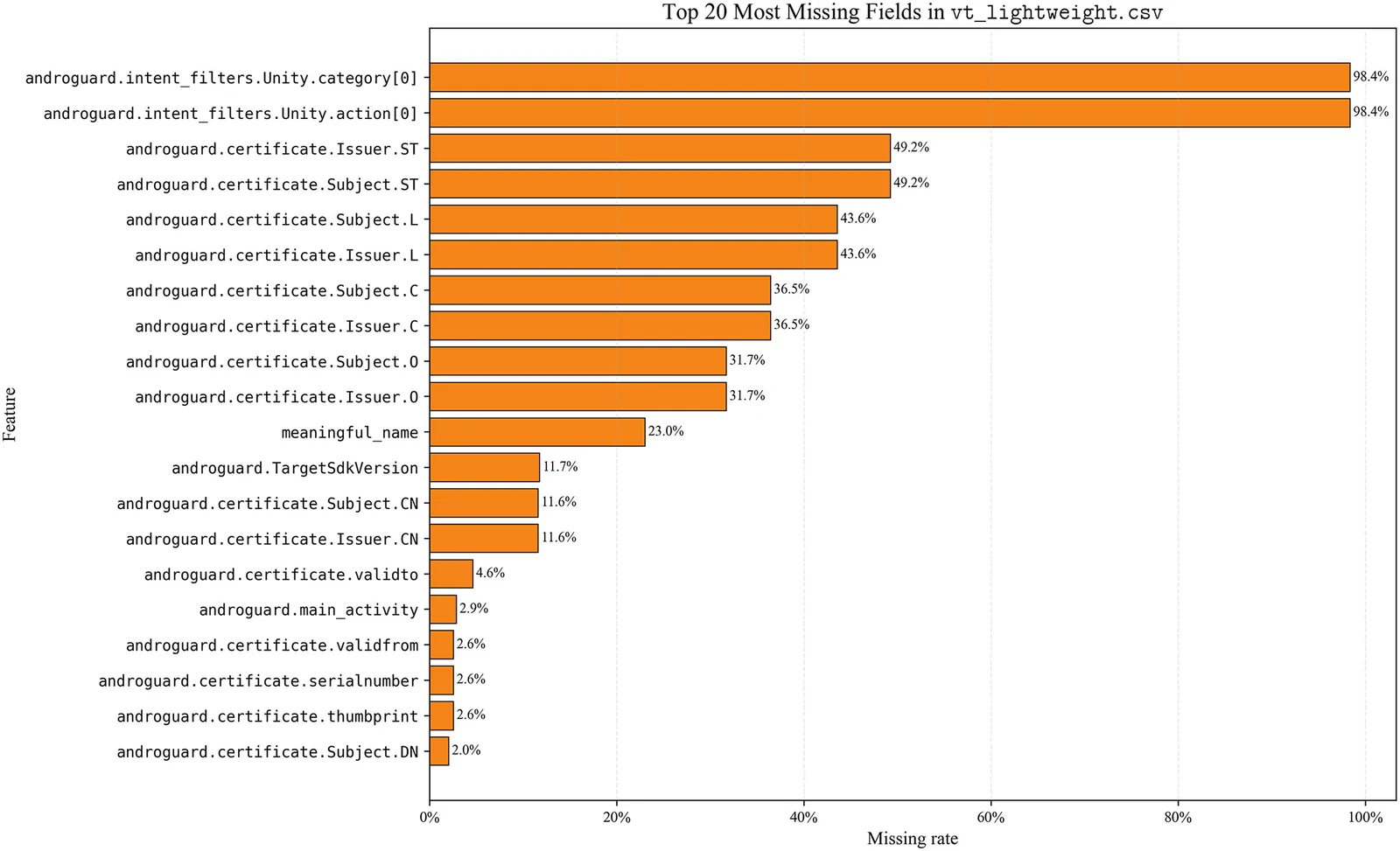
Top 20 most-missing fields in the VirusTotal lightweight table. The sparsest fields are androguard.intent_filters.Unity.category[0] (98.36%) and androguard.intent_filters.Unity.action[0] (98.35%), followed by certificate metadata such as Issuer.ST and Subject.ST (both about 49.23%).

Fig. 4 highlights the 20 most-missing fields in the VirusTotal lightweight table. Optional Unity intent-filter fields and certificate location fields are the sparsest attributes, whereas the core identifiers used in the released summary dataset are substantially more complete.

**Fig. 5:**
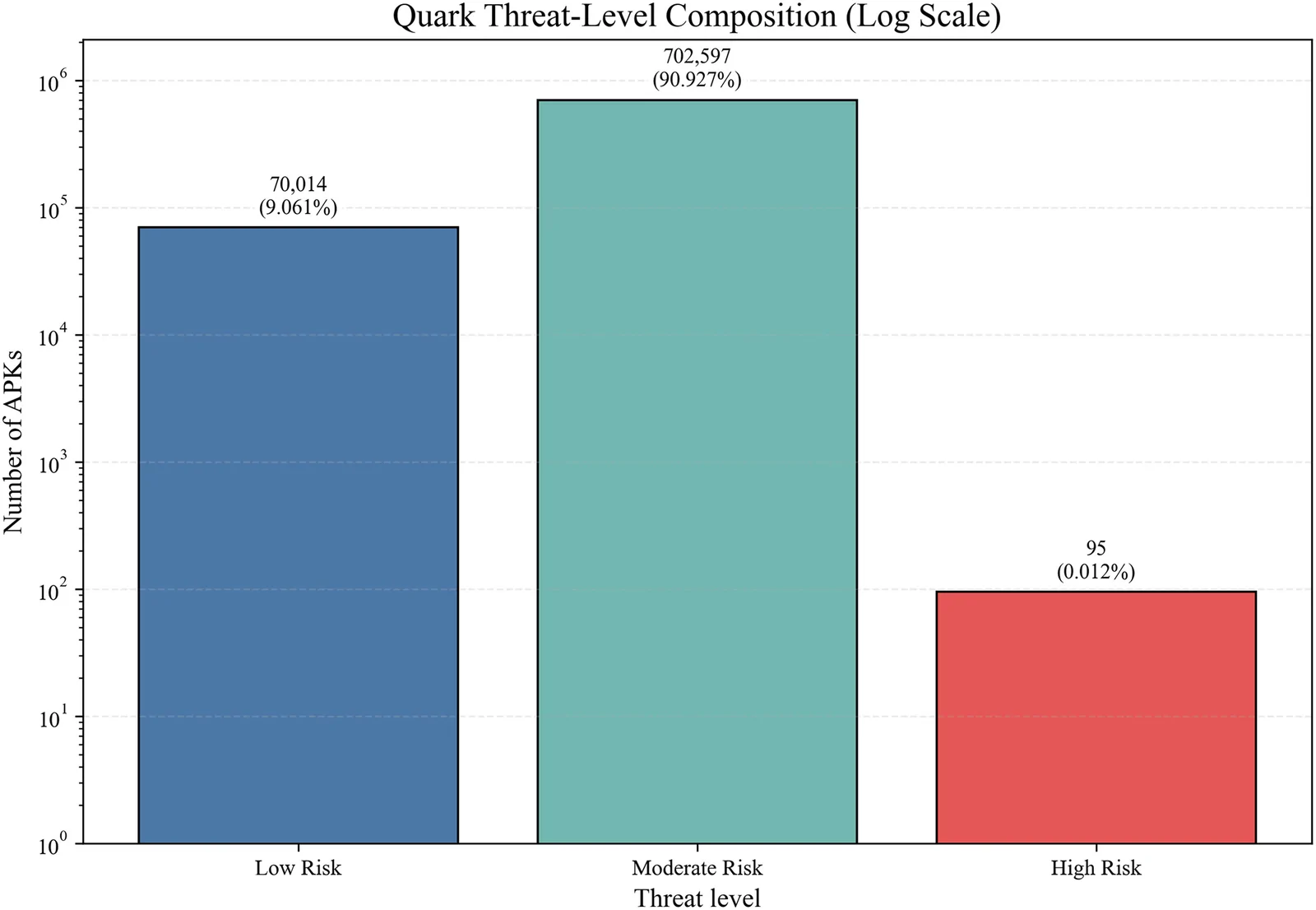
Threat-level composition in the Quark-Engine extensive dataset. Quark-Engine labels 702,597 APKs (90.9%) as moderate risk, 70,014 (9.1%) as Low Risk, and 95 as high risk.

Fig. 5 shows the Quark-Engine threat-level composition. The dataset is dominated by the Moderate Risk class, which is important to keep in mind when using raw Quark labels directly.

**Fig. 6:**
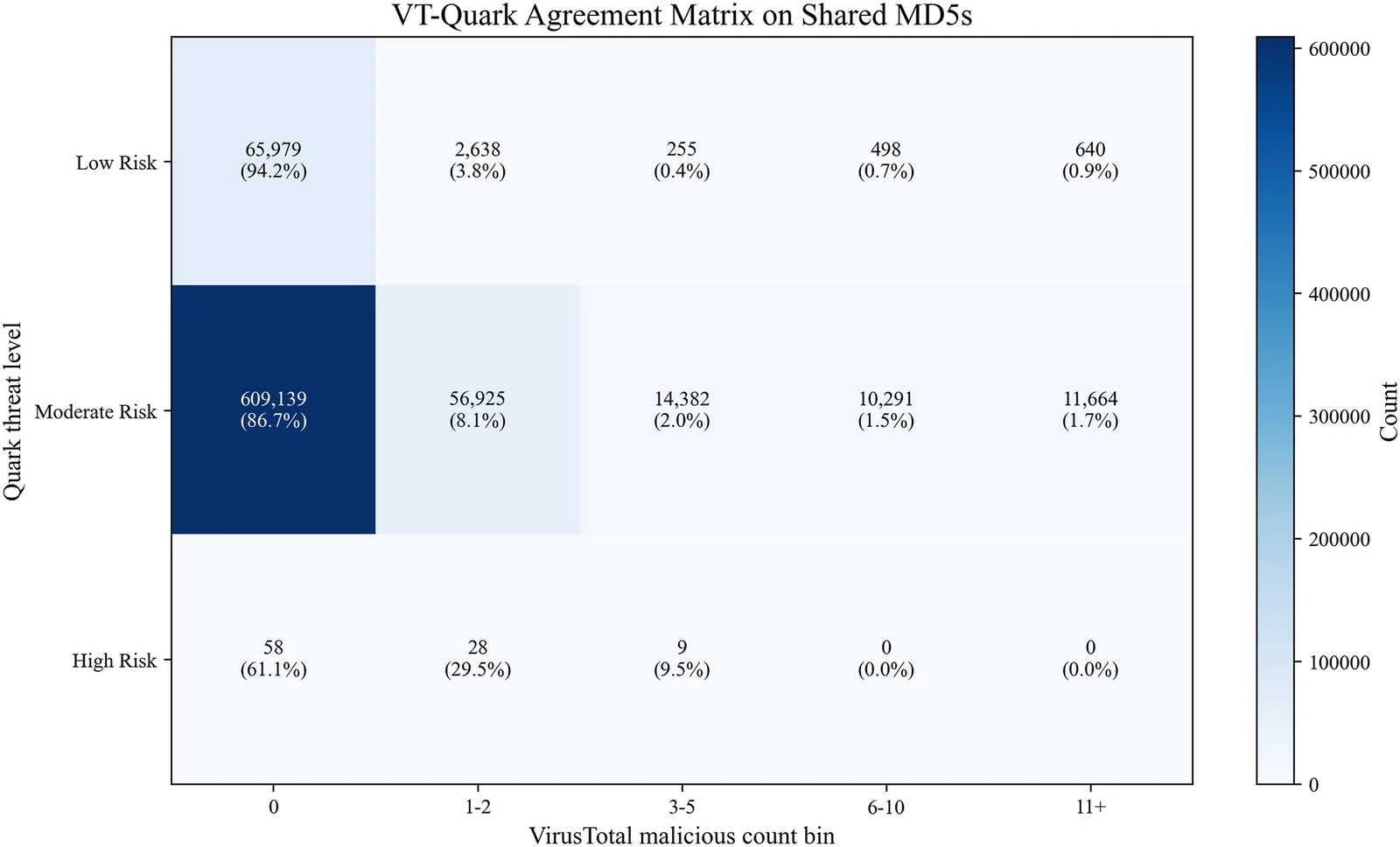
Agreement matrix between Quark-Engine threat levels and binned VirusTotal malicious counts on shared MD5s. The dominant Moderate Risk row still has 609,139 APKs (86.7%) in the VirusTotal zero-detection bin, while the distribution shifts gradually toward higher VirusTotal bins as Quark severity increases.

Fig. 6 relates Quark-Engine threat levels to binned VirusTotal malicious counts on the shared MD5 intersection. The figure shows partial agreement rather than a one-to-one mapping: higher Quark severity tends to shift samples toward higher VirusTotal bins, but most *Moderate Risk* apps still fall into the VirusTotal zero-detection bin.

The overall exact agreement between binarized Quark threat levels (Low Risk = benign, Moderate/High Risk = malicious) and VirusTotal threshold k = 1 labels is 20.6% (Cohen's κ = 0.015, slight agreement), confirming that the two sources provide strongly divergent signals (Quark labels 90.9% of apps as malicious versus 12.6% for VirusTotal). This disagreement, visualized in Fig. 6, highlights the complementary nature of static rule-based analysis (Quark) versus multi-engine reputation (VirusTotal), and directly motivates the three-source consensus validation procedure used to construct the benchmark subsets in Table 3.

### 3.4 Validated subsets

The validated subsets are created after excluding apps submitted only once to VirusTotal and then requiring agreement among the original label, the VirusTotal threshold rule, and Quark-Engine. This pre-filter leaves 473,668 valid candidate apps. Table 3 reports the class composition and dimensions of each validated subset.

As the VirusTotal threshold increases, the validated subsets become smaller but progressively more balanced. This gives users a direct and transparent way to choose a benchmark that matches their preferred trade-off between sample count and label strictness.

Table 5 positions our dataset against three widely used Android malware benchmarks. While DREBIN offers a large feature set, it relies on static analysis alone and is not openly licensed. AndroZoo provides scale but uses only VirusTotal labels and lacks security-specific features. CIC-AndMal2017 includes dynamic analysis but is an order of magnitude smaller [12]. Our dataset bridges these gaps by combining scale (772k samples), three-source consensus labeling (original removal status + VirusTotal + Quark-Engine), 59 analytical features, and five ready-to-use validated subsets under an open CC BY 4.0 license.

**Table 5:** Comparison of the proposed dataset with existing Android malware benchmarks.

Feature	DREBIN [11]	AndroZoo [3]	CIC-AndMal2017 [12]	Ours [13 ]
Number of applications	∼556,000	∼10,000,000	∼5000	**772,533**
Label source	Static analysis only	VirusTotal (single threshold)	Dynamic + static	**Three-source consensus**
Number of features	∼8000 (binary)	Metadata only	∼80	**59 (incl. 10 security features)**
Multi-source labels	No	No	No	**Yes (3 sources)**
Validated subsets provided	No	No	No	**Yes (5 thresholds)**
Open license	No	Yes	Yes	**Yes (CC BY 4.0)**
Reproducibility documentation	Limited	Limited	Moderate	**Full coverage reporting**

The ∼556,000 figure for DREBIN refers to the feature matrix dimension; the original DREBIN dataset contains approximately 130,000 applications [11].

## 4 Experimental Design, Materials and Methods

### 4.1 Data collection

The starting point is the longitudinal Google Play removal dataset of Mohsen et al. [1,2], containing 870,515 Android applications. For each APK, we attempted to collect two external analysis products: a VirusTotal Premium v3 Enterprise report retrieved by MD5 hash through the v3 API [10] and a local Quark-Engine 25.3.1 report generated from the APK file [9].

For every application hash in the original dataset, we queried the VirusTotal v3 API between March and May 2025, successfully retrieving 803,320 reports. Each VirusTotal report aggregates verdicts from approximately 70 antivirus engines. Quark-Engine 25.3.1 was executed locally on all accessible APKs between March and April 2025, producing 772,732 analyses. Quark-Engine 25.3.1 was executed using its default scanning rule set and confidence weightings, with no custom rule modifications applied. Quark collection used a two-stage timeout policy: 2 min initially, then 10 min for previously skipped APKs.

After merging VirusTotal and Quark-Engine results by MD5 hash, we obtained 772,533 applications with complete paired analyses. The difference from the original 870,515 samples arises primarily from API timeouts, missing hashes, and unprocessed APKs that exceeded scan limits. All processing was performed on a Linux workstation (AMD EPYC 7313, 64 GB RAM) using Python 3.10.

The size distribution of successful and failed Quark scans reveals a clear collection difficulty. APKs that completed within the 10-minute limit had mean size 10.476 MB, whereas APKs that still timed out had mean size 16.159 MB, a 42.7% increase. This large gap indicates that file size was an important practical constraint for Quark-Engine scanning in this corpus.

After both collection stages, we intersected the VirusTotal and Quark-Engine results on MD5 and obtained 772,533 paired applications. These paired apps form the basis of the summary dataset, the extended paired dataset, and the validated subsets.

### 4.2 Data cleaning and normalization

The raw JSON responses were parsed into stable tabular schemas. For the VirusTotal extensive dataset, we retained 55 attributes chosen to balance informativeness, completeness, and reproducibility. We deliberately excluded highly volatile per-engine verdicts, redundant identifiers, and very high-cardinality raw fields that would not transfer well across future datasets. Of the 55 retained VirusTotal attributes, 53 were extracted directly from the structured JSON responses. The two launcher-related intent-filter fields were recorded only when the VirusTotal/Androguard response indicated a launcher activity, which keeps these fields comparable across apps.

VirusTotal missing values were normalized as follows: missing numeric values were filled with −1, while missing categorical, textual, list, and date-like values were filled with None. We observed that 45,243 apps, or 5.632% of the 803,320 VirusTotal rows, contain all 55 retained VirusTotal attributes. Missingness is concentrated in optional Unity fields and certificate Distinguished Name subfields. In the compact 5-feature VirusTotal summary, 736,780 of the 772,533 paired apps (95.372%) contain all five selected VirusTotal features. The main missing derived field is certificate_life_days, absent in 35,753 paired apps because one or both certificate date endpoints are missing.

For the Quark-Engine extensive dataset, we retained 12 attributes that summarize file metadata, overall risk, and rule-level evidence. Empty permission lists were treated as observed empty lists rather than as missing values. The Quark JSON parser successfully yielded all 12 attributes for 772,320 of 772,732 rows (99.946%). Due to parsing failures, 27 apps were skipped during detailed tabulation. Among the retained rows, the remaining missing fields are very rare, with counts ranging from 296 to 339 depending on the specific crimes.* field. In the compact 5-feature Quark summary, all 772,533 paired apps contain all five selected Quark-Engine features.

After parsing and normalization, the final processed tables were written in UTF-8 CSV format using consistent column naming. The release includes the compact summary table, the extended paired table, and the validated subsets so that users can reproduce every aggregation step.

Feature selection justification. The five VirusTotal features were selected based on three criteria: (a) completeness rate exceeding 95% across paired samples, (b) direct analytical relevance to malware detection (e.g., malicious_count, undetected_count), and (c) avoidance of redundant or highly collinear fields. The four Quark-Engine features were selected similarly.

### 4.3 Validation procedure

We define an application as *benign* if all three sources agree:

• original label = non-removed;
• Quark-Engine threat level = low;
• VirusTotal malicious count = 0.

We define an application as *malicious* if all three sources agree:

• original label = removed;
• Quark-Engine threat level ∈{moderate,high};
• VirusTotal malicious count ≥k, where k∈{1,2,3,4,5}.

Before applying these rules, we excluded apps with times_submitted =1 in VirusTotal in order to focus on files seen multiple times by the platform. This reduces the candidate pool to 473,668 apps. Applying the consensus rules to that pool yields the five validated subsets listed in Table 3.

## Limitations

Approximately 11.3% of the original corpus could not be retained in the final paired release because of unavailable files, query failures, timeouts, or other processing gaps. The enriched labels are based on static-analysis-oriented sources only (VirusTotal multi-scanner and Quark-Engine rule-based analysis). Dynamic runtime behaviors—such as obfuscated code execution, environment-triggered payloads, or reflective loading—are not captured, which may underrepresent certain malware families. Users requiring behavioral features should complement this dataset with dynamic analysis tools.

*Time dependence.* VirusTotal was queried in 2025 for APKs originally submitted in 2016–2017. Retrospective signature updates by antivirus engines may have inflated detection counts compared to real-time 2017 scanning. Similarly, Quark-Engine outputs reflect version 25.3.1; future tool versions may produce different results. Researchers performing longitudinal studies should account for this temporal drift.

*Label strictness vs. coverage.* The validated subsets require three-source consensus (original label, VirusTotal threshold, Quark-Engine threat level). This increases label quality but reduces coverage: only 8.1–17.0% of the 473,668 candidate apps are retained depending on threshold (Table 3). Users should note the substantial **class imbalance** in these subsets (e.g., at k = 1: 18,275 benign vs. 62,093 malicious). Stratified sampling, resampling, or cost-sensitive learning is recommended for classifier training.

*Linkage and data gaps.* MD5 is used as the sole linkage key between dataset components. MD5 is cryptographically weak and collisions are theoretically possible, especially for repackaged malware; future versions should employ SHA-256. Of the 772,732 Quark-Engine reports, 27 apps (0.054%) failed parsing with no systematic pattern identified; these are excluded.

*Raw JSON data.* The raw VirusTotal and Quark-Engine JSON responses are not publicly available due to their large volume (>500 GB) and API terms of service. They are retained privately by the authors and available upon reasonable request. Researchers may re-query VirusTotal using the provided MD5 list and documented methodology.

## Conclusion

This data article presents a large-scale Android application dataset enriched with VirusTotal and Quark-Engine intelligence. The dataset contributes: (1) a three-source consensus labeling framework combining original Google Play removal status, VirusTotal multi-engine verdicts, and Quark-Engine static analysis; (2) nine derived security features (five from each source) that eliminate months of API querying for downstream users; (3) five validated benchmark subsets at VirusTotal thresholds k = 1 through k = 5, allowing researchers to trade dataset size (38,302 to 80,368 samples) against label strictness; and (4) transparent reporting of coverage gaps, missingness, parsing failures, and cross-source disagreement.

Primary use cases include malware detection model training, label-noise sensitivity analysis, and reproducible benchmarking. Future work will focus on three directions: migrating from MD5 to SHA-256 for collision-resistant linking, integrating dynamic analysis features to capture runtime behaviors, and periodically re-querying VirusTotal to study label evolution over time. The dataset is publicly available under CC BY 4.0 license at https://doi.org/10.34894/XV84Z8.

## Ethics Statement

The authors confirm that they have read and follow the ethical requirements for publication in Data in Brief and confirm that the current work does not involve human subjects, animal experiments, or any data collected from social media platforms.

## CRediT Author Statement

**Yulin Zhang:** Conceptualization, Data curation, Methodology, Software, Validation, Writing – original draft. **Ali Murat Gültekin:** Investigation, Data curation, Visualization. **Halil Bisgin:** Conceptualization, Resources, Supervision. **Koerner Buchta:** Conceptualization, Methodology. **Fadi Mohsen:** Conceptualization, Project administration, Supervision, Writing – review & editing.

## Data Availability

Scripts (Original data)
DataverseAndroid Datasets: Integration of VirusTotal and Quark-Engine Intelligence (Original data)
